# Policy dialogue and participation: a new way of crafting a national health financing strategy in Morocco

**DOI:** 10.1186/s12961-020-00629-2

**Published:** 2020-09-29

**Authors:** El Houcine Akhnif, Hafid Hachri, Abdelouahab Belmadani, Awad Mataria, Maryam Bigdeli

**Affiliations:** 1 World Health Organization Country Office of Morocco, 3 Avenue S.A.R. Sidi Mohamed, Rabat, Morocco; 2 Ministry of Health, Directorate of Planning of Financial Resources, 335, Avenue Mohamed V, Rabat, Morocco; 3 World Health Organization Regional Office for the Eastern Mediterranean, PO Box 7608, Nasr City, Cairo 11371 Egypt

**Keywords:** Health financing, Universal Health Coverage, policy dialogue, collaborative approach

## Abstract

**Background:**

Policy dialogue for health policies has started to gain importance in recent years, especially for complex issues such as health financing. Moroccan health financing has faced several challenges during the last years. This study aims to document the Moroccan experience in developing a consolidated health financing strategy according to the policy dialogue approach. It especially considers the importance of conceptualising this process in the Moroccan context.

**Method:**

We documented the process of developing a health financing strategy in Morocco. It concerned four steps, as follows: (1) summarising health financing evidence in preparation of the policy dialogue; (2) organising the health policy dialogue process with 250 participants (government, private sector, NGOs, civil society, parliamentarians, technical and financial partners); (3) a technical workshop to formulate the strategy actions; and (4) an ultimate workshop for validation with decision-makers. The process lasted 1 year from March 2019 to February 2020. We have reviewed all documents related to the four steps of the process through our active participation in the policy debate and the documentation of two technical workshops to produce the strategy document.

**Results:**

The policy dialogue approach showed its usefulness in creating convergence among all health actors to define a national shared vision on health financing in Morocco. There was a high political commitment in the process and all actors officially adopted recommendations on health financing actions. A strategy document produced within a collaborative approach was the final output. This experience also marked a shift from previous top-down approaches in designing health policies for more participation and inclusion. The evidence synthesis played a crucial role in facilitating the debate. The collaborative approach seems to work in favouring national consensus on practical health financing actions.

**Conclusion:**

The policy dialogue process adopted for health financing in Morocco helped to create collective ownership of health financing actions. Despite the positive results in terms of national mobilisation around the health financing vision in Morocco, there is a need to institutionalise the policy dialogue with a more decentralised approach to consider subnational specificities.

## Key messages of the paper

- Policy dialogue approaches legitimise the adoption of a health financing strategy.
- Participation in designing health financing strategies is important for a shared vision on health financing.
- Policy dialogue for health financing strategies needs to be institutionalised and cover sub-national levels.

## Introduction

Universal Health Coverage (UHC) is an opportunity to strengthen health systems and promote equity [1]. Countries need to develop a concrete health strategy to achieve health system objectives and targets by moving away from political discourse and making UHC a reality. UHC is a powerful means to achieve the Sustainable Development Goals, where health system strengthening becomes mandatory to improve health indicators and provide health for all while leaving no one behind [2]. In many low- and middle-income countries (LMICs), pathways towards UHC imply introducing and rolling out medical coverage schemes, particularly for under-covered groups such as impoverished households and people working in the informal sector [3]. In this sense, health system strengthening becomes essential in concretising UHC [1, 4].

By definition, UHC is the capacity to provide all people with access to health services of sufficient quality, while also ensuring that the use of these services does not expose the user to financial hardship [5]. For UHC policies to truly respond to the population needs, an accountability framework is required as part of a social contract between citizens and the state. This social contract is essential to give a voice to citizens and build a new relationship between healthcare, medical innovation systems and society, which determines the sustainability of health systems [6]. There is a crucial link between social protection politics and the way social contracts are built in countries, especially in Africa [7]. Liberal or social policy choices will have implications on social protection and on whether participation is part of the formulation process. In Morocco, social protection has always been present as a means to legitimate policies, yet started to gain importance after the Arab spring social movements. For example, since independence in 1956, the government applied free services at the level of primary healthcare and free services for the poor at the level of hospitals.

Health financing is one of the six blocks of the health system [8]. There are tools and theories on how to design health financing strategies from the technical perspective [9–13]. However, health systems are complex by nature and experiences cannot be simply transferred without adaptation [14–16]. Unfortunately, a generic approach to formulating health financing strategies does not exist; therefore, mixing learning from other countries’ experiences and strengthening national adaptation mechanisms is essential. In this way, adopted strategies will consider the specificities of the context and will be owned by relevant stakeholders. Furthermore, learning and adaptation are critical to reaching a shared vision and one way of doing this is through creating spaces for policy dialogue [17–20].

The policy dialogue is defined as “*… part and parcel of policy and decision-making processes, where they are intended to contribute to developing or implementing policy change following a round of evidence-based discussions/workshops/consultations on a particular subject*” ([21], p. 328). Fruitful policy dialogue is the one that provides an opportunity to debate considering different options for discussion; it should chart a way forward in terms of how the outcomes will be implemented and its content should be shared and timely, in accordance with the culture or norms of the community, and be seen as a priority by all stakeholders [21]. It is also an iterative process that includes both technical and political aspects of the problem in question; it uses evidence-based and politically sensitive discussions, it is inclusive for a range of key stakeholders, and it has a clear purpose or outcome in mind, e.g. a decision, a plan or a deliverable. Moreover, a policy dialogue should be based on leadership, flexibility, ownership, relationship-building, collaboration, communications, information sharing, transparency and trust [22].

Policy debates at the national level on how a country wants to design its health financing system will give chances to succeed in implementing health strategies. Indeed, in any effective strategy, mobilisation of all actors at different design stages and levels is needed, especially for the implementation phase that requires a long-term commitment. If the policy dialogue has shown good results in developed countries, LMICs will be even more in need of it. To accompany policies in all stages (agenda-setting, policy formulation, policy implementation, policy evaluation), the institutionalisation of dialogue is crucial for the continuity and maintenance of institutional intelligence [21, 23].

The path to universality in Morocco was designed around three components, as follows: a compulsory health insurance scheme for formal employees of both private and public sectors (it covers 34% of the population); the scheme for the poor and vulnerable (*Régime d’Assistance Médicale* or RAMED) has contributed to improving the coverage rate to reach 62%, according to the latest figures; and the scheme for self-employed, whose law was passed by the parliament on June 23, 2017. The latter is the most challenging in terms of implementation as it concerns the informal sector and highly heterogeneous categories of the population with less capacities to contribute.

Different studies highlighted the challenges of health financing in Morocco. Whilst the compulsory health insurance scheme (*Assurance Maladie Obligatoire*) financing presents fewer challenges [24], the scheme for the poor (RAMED) is still struggling to achieve its objectives. The RAMED was generalised since 2012 and always strives to ensure equity of access to health services, reducing financial hardship and providing sustainable financing for the scheme [25, 26]. Another study using longitudinal data from 2013 to 2015 showed an increase in household expenditure associated with the generalisation of RAMED [27]. According to national health accounts in Morocco, in 2013, the total health expenditure reached ~52 billion Dirhams (US$6 billion at the 2013 exchange rate). The per capita health expenditure is nearly US$188. The total health expenditure represents 5.9% of the GDP. The health system financing sources are the tax revenue (24.4%), households (50.7%), health insurance (22.4%), employers (1.2%), international cooperation and others (1.3%). The collective health financing is still limited (48.8%) and households cover more than half of total health expenditure out of pocket [28]. The high household expenditure could partly be explained by high self-medication, the low health insurance coverage (62%), the resource allocation process and the insufficiency of hospital budgets.

These studies and many others stressed the difficulties that Moroccan health financing is experiencing in many areas. Indeed, there are, among others, challenges related to persisting passive purchasing (the purchasing relation with fewer incentives to improve the health provider’s performance), the need to extend the coverage, the need to link the benefits package definition to health financing capacity, and the fragmentation of pooling. To deal with these challenges, Morocco decided to formulate a consolidated health financing strategy using a national dialogue approach.

This paper documents the conceptualisation and application of the policy dialogue on health financing in Morocco. The Global Action Plan initiative for Healthy Lives and Well-being for All [29] and the national cooperation of multiple partner agencies supported the policy dialogue. This process started in March 2019 and finished in February 2020 with the finalisation of a draft health financing strategy awaiting final Ministerial endorsement.

## Methods

### Main data sources and data analysis

The main data sources for this documentation are (1) mission reports and presentations of the first scoping mission on health financing organised by WHO, (2) the detailed reports of each workshop prepared by the teams of rapporteurs during the national conference as well as the reports of both follow-up workshops, (3) the presentations that were made by national and international experts, decision-makers from other countries (ministers and experts), (4) the final report of the policy dialogue recommendations, (5) notes from our observation as participants and organisers, and (6) health financing documentation review prepared as the background of the policy dialogue.

All reports and documents were centralised and categorised according to each step of the process (preparatory phase including health financing documents review, the policy dialogue conference documents, presentations and reports, and the two workshop minutes summarising the discussions). The analysis was performed in such a way as to extract details about the whole process of the policy dialogue from evidence gathering to the strategy draft finalisation. Based on the detailed reports of sessions and workshops, data were charted using a grid that follows the different phases of the policy dialogue.

We then used a coding system to chart the data in a way to reconstruct each step of the process. This qualitative coding allowed structuring of the information and its analysis according to the specificity of each phase. As most of the policy dialogue steps were documented, the quality of findings was checked by sharing and cross-checking the results among the researchers, who themselves contributed and participated in the whole process. When missing parts are identified, we went back to the source documents and completed the analysis.

### Conceptual framework

**Fig. 1:**
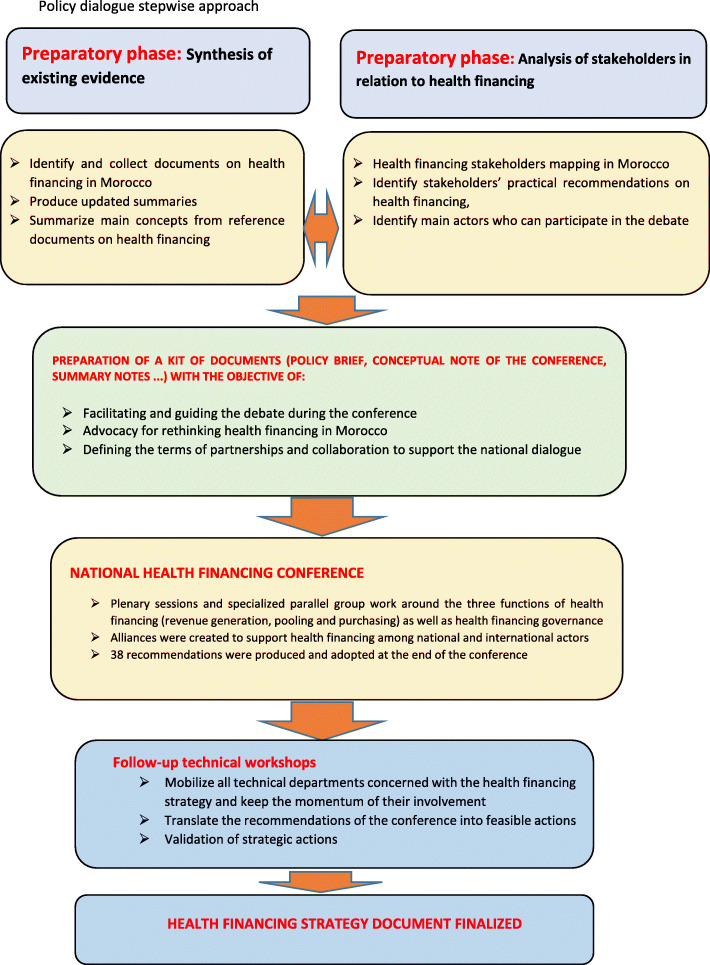
Policy dialogue stepwise approach

A stepwise approach was used to structure the process of developing national recommendations for a health financing strategy in Morocco. The stepwise approach allowed understanding of the current status of health financing in Morocco and the consensus of national stakeholders on future visions and directions. This stepwise approach is described in Fig. 1.

### Participation

Participation in the different steps of the policy dialogue was organised based on criteria related to the involvement in health issues, either directly or indirectly, through actions on health determinants. An inclusive list was established within the organisation committee (four technical agencies: WHO, European Union, World Bank and African Development Bank, and members from the Ministry of Health (MoH) and from other non-governmental departments).

The policy dialogue involved the following participants:

- Prime minister, other ministers, former ministers and government representatives
- Parliament representatives of the social commission
- National observatories, Ministry of Planning
- Technical and financial partners, international experts and foreign ministers of health (Turkey and Senegal)
- National professional councils, national experts from research centres and national institutes and representatives of medical schools
- Representatives of the private sector associations
- Trade union representatives
- Civil society organisations with an active role in health
- Representatives of the health insurance agency and health insurance funds
- Representatives of hospitals, including teaching hospitals
- Professional associations
- Media and analysts in the social fields

The first follow-up workshop involved 22 experts from national departments concerned with health financing to translate the outputs of the policy dialogue into feasible actions. The second follow-up workshop involved decision-makers in the field of health financing (departments of the MoH, health insurance funds, Ministry of Finance, an external expert in health financing), including technical and financial partners (WHO, European Union, World Bank and African Development Bank) as a commitment to support the implementation phase of the strategy.

### Preparatory phase

The authors of this paper participated and contributed to the whole process (design, management and documentation of the process). All preparatory meeting minutes, documents and workshop reports were analysed to redesign the framework of the policy dialogue for further learning. Each step of the health financing debate in Morocco was structured and organised to ensure the participation and inclusion of all actors.

During the preparatory phase of the policy dialogue, technical staff from the MoH and WHO reviewed and summarised essential documents on health financing to facilitate the debate. These were research studies and consultant reports produced in Morocco between 2013 and 2018. The objective of this review was to provide access to available evidence on health financing in Morocco to all stakeholders involved in the policy dialogue process.

Before the conference, a series of discussions with the main actors in the Moroccan health system allowed the mapping of the challenges around which the national debate would be organised. During these discussions, additional documents were also identified for the review. The preparatory phase was also an opportunity to identify the main actors to be invited to the national forum.

The policy dialogue benefited from the collaboration between WHO, the European Union delegation in Morocco, the African Development Bank and the World Bank. Each of these organisations had, in recent years, produced evidence and provided technical assistance on different components of health financing in Morocco (e.g. study of catastrophic health expenditure, provider payment methods, etc.) The collaboration allowed sharing of the evidence and technical expertise and the mobilising of experts and representatives from different countries to share country experiences and best practices during the dialogue process.

The MoH steered the entire dialogue process, including the collaboration of partners and the selection, synthesis and dissemination of available information.

### The national conference on health financing

The national conference on health financing was held on June 18–19, 2019, in Rabat; around 250 participants contributed to this event. The participation covered all actors concerned by the health system (see above). Different ex-ministers from other countries and international experts in the field of health financing shared their experiences. Two plenary sessions focused on sharing national and international expertise on health financing and health financing governance. Three parallel workshops were held to deepen the dialogue on health financing functions (mobilisation of resources, pooling and purchasing). The parallel workshops were designed to give more time for debate and participation. The organisation committee designated a general rapporteur who was responsible for consolidating the overall proceedings of the conference as well as rapporteur teams and moderators for each session. Templates were used for rapporteurs to document each step of the national event, including guiding questions for facilitators. Box 1 gives an example of the guiding questions for the resource mobilisation workshop.

### Technical workshops to develop the strategy

As a follow-up to the national conference, two workshops were organised to translate the health financing recommendations adopted during the debate into vision, strategic directions and feasible actions. Members of the research team participated in these workshops and documented the discussions. These two workshops were held by WHO to identify the main challenges and explore all current and future windows of opportunity to define concrete actions for implementation. The first workshop was technical, with the participation of professional staff and managers representing all departments involved in health financing. The second workshop was aimed at decision-makers’ validation of the health financing actions and to discuss how partners could support the implementation phase.

Box 1 Guiding questions for the resource mobilisation workshop What is the situation in Morocco in terms of resource mobilisation for health? How does Morocco compare to other countries?

• What are the existing constraints for an optimised resource mobilisation process for health?

• What opportunities exist in terms of fiscal space for mobilising resources for health?

• How can civil society organisations and parliamentarians contribute to the debate and enrich the discussion towards a national consensus on a vision on resource mobilisation for health?

• What are the existing possibilities for additional mobilisation of resources for health, in particular, innovative financing, partnership, etc.?

• What recommendations can each stakeholder make in this regard? What is the point of view of experts in relation to the proposals for the case of Morocco?

• What concrete actions can be taken for the short-, medium- and long-term vision in terms of mobilising resources for health?

## Results

The results of this paper are structured around key components of the policy dialogue process, namely a summary of existing evidence on the current situation in Morocco, political commitment, participation, and exchange of experiences, the health financing functions and their governance. The following sections present the findings following each step of the policy dialogue.

### Preparatory phase

#### Summary of existing evidence on the current situation in Morocco

A summary of existing evidence on the current situation in Morocco was shared with participants. This summary was organised into three groups. The first synthesis provided an overview of the Moroccan health system using the latest data and health indicators. The health system overview also presented the organisational aspects and main challenges that Morocco is experiencing (coverage, governance, human resources, etc.). The second synthesis was about the health financing analysis and indicators as well as its main challenges based on national health accounts and recent studies produced at the national level. In this synthesis, the financing of UHC schemes was examined by stressing areas of success but also pitfalls. Recent studies on the fragmentation and payment mechanisms were summarised, emphasising the difficulties that the purchasing function is facing. The third synthesis was about studies on the benefits package and access to health services, especially for the vulnerable population and former strategies and reforms. These syntheses covered financial access barriers and the quality of care and its link to health financing. International experiences were summarised and covered health financing strategies in general and, more specifically, areas of strategic purchasing in different countries (Germany, France, Turkey and others). These international experiences contributed to enrich the debate by stressing the implementation challenges for each strategy. These syntheses were presented at the beginning of each workshop to give participants insights on what worked well in other settings, including a summary of technical recommendations for health financing.

### The national conference on health financing

#### A high political commitment and participation

The health financing dialogue organised in June 2019 benefited from the support of His Majesty, the King, through the Royal patronage. The Head of Government (the Prime Minister) as well as other ministers from the government supported the national debate through their active participation. The importance of the dialogue was reflected in massive national media attention [30]. The debate was supported by all three levels of WHO [31]. Participation in the conference was also an important indicator to assess national dialogue success. Three months later, during the UN General Assembly in New York in September 2019, the Minister of Foreign Affairs, jointly with the MoH, organised a side event to share the Moroccan experience on the health financing dialogue [32]. The side event was well attended by ministers of health and foreign affairs of many African countries.

#### Learning from national and international experiences

Former ministers of health of other countries that achieved excellent results in terms of health financing came to present their successes during the national conference but also stressed the challenges faced in implementation and equity (ex-ministers of health of Turkey and Senegal). Other examples included France and Ghana and emphasised strategic aspects in the area of health financing. International examples focused on the importance of promoting primary healthcare through incentives from health financing and the convergences of UHC schemes to ensure more equity in the health system. The presentation of countries’ path to UHC gave different perspectives for Moroccan participants to think of the diversity of approaches of a health financing strategy. The discussions also covered the roles, commitment of actors, central place of the benefits package and how they link to health financing. The financial protection and all measures adopted in each country were essential in the sharing of these experiences. International experts and resource persons also addressed the role of the private sector in the path to UHC and strategic purchasing as a leverage to improve the overall health system performance through health financing payment mechanisms. Former ministers of health discussed the complexity of the policy design and implementation and shared their specific approaches with Moroccan policy-makers. The Moroccan health system and its main challenges and achievements were presented during this session to give a background for international experts.

#### Resource mobilisation

The resource mobilisation workshop tackled the main challenges in Morocco and debated the following elements: (1) the situation regarding the resource mobilisation function for health in Morocco; (2) the mobilisation of extra-budgetary financial resources for the health sector; (3) main successful mechanisms to expand fiscal space for health; (4) taxation as an innovative source of financing in the health sector, and (5) practical recommendations to improve the mobilisation of resources for health. Participants acknowledged the effort of the state in terms of mobilising resources for health (the budget of the MoH moved from US$1.2 billion to US$1.9 billion between 2012 and 2020 [33, 34]). They analysed the main challenges, such as the escalation of health services costs, the increasing needs of the population in terms of access to health services and the low self-financing capacity of health facilities. To stimulate the debate, international experts presented specific examples from different countries, notably that of Rwanda (Community Health Insurance), Mexico (Seguro Popular) and Argentina (Plan Nacer), and the importance of fiscal space to support human development objectives. International experts supported the debate to bring lessons from the global level but also explained macroeconomic conditions, sources of domestic income and redefinition of priorities. Box 2 presents the strategic recommendations for the Resources mobilisation function.

#### Pooling of resources

The dialogue around the pooling of resources in Morocco focused on reducing fragmentation between existing schemes. National experts presented the Moroccan experience and discussed its details with participants. Multiple insurance schemes cover portions of the population in Morocco, including private-sector employees, civil servants, students, self-employed workers, internal plans for some large companies, and the RAMED subsidies [24]. These multiple funds are disconnected, without solidarity between them. International experts presented different experiences regarding the pooling function and how it is designed in various settings. Experts from Ghana and WHO presented lessons and discussed recommendations from their perspectives. The main ideas of these experiences were increasing solidarity through pooling, reducing fragmentation and having the capacity to cover a comprehensive benefits package. All participants agreed that a health financing strategy should ensure a progressive convergence in the long term towards a unified scheme. The existence of several schemes generates high management costs. Box 3 presents the strategic recommendations for the Pooling function.

#### The Purchasing function

The workshop related to the Purchasing function raised the issue of the benefits package and the difficulties in quantifying its services. In Morocco, the benefits package is defined in broad terms and mainly through a description of activities [35]. The analysis of the Moroccan example, as compared to international practice, raised the importance of revising the benefits package in different ways. Instead of defining it broadly around activities, the discussion recommended to illustrate it around diseases to facilitate the estimation of cost and required financing. Participants raised the importance of increasing public hospital’s autonomy as a ground of performance-based funding or contracting mechanisms. Participants also debated the importance of building strong partnerships with the private sector based on performance improvement vision through contracting. The issue of purchasing for UHC brought up the importance of creating incentives through payment mechanisms to influence the distribution of health providers all over the country.

It was highly recommended to consider the needs of vulnerable groups in the design of the benefits package but also through contracting mechanisms. The objective is to ensure that no one is left behind and the Purchasing function enables the implementation of incentives that support operationalising this value in the health system. Box 4 presents the strategic recommendations for the Purchasing function.

#### Governance of health financing

The plenary session devoted to health financing governance started by confronting the Moroccan experience with other countries’ and the analysis of experts from WHO and the European Union. Discussions revolved around the importance of working on the unification of all health insurance schemes, mainly through the same reimbursement rates and benefits package, as well as the creation of an independent fund for the management of RAMED resources. Creating regional committees for decisions on health financing was highlighted as a requirement to improve the governance function. The separation of functions (funding and health provision) was stressed as a strategic requirement for UHC in general and particularly for RAMED to sustain in the future. An essential part of the debate focused on governance entities and their roles like the National Agency of Health Insurance (*Agence Nationale de l’Assurance Maladie*) and the inter-ministerial committee that should be strengthened. The role of evidence for improving governance was also discussed to recommend improving the UHC information system. The autonomy of regions and empowering them with regional policy dialogues was mentioned as a determinant for better governance. Another element concerned the creation of spaces and channels for dialogue and participation of stakeholders, including the population, to develop recommendations for decision-makers. These spaces of discussion should be inclusive of all actors involved in health (private sector, government, civil society organisations, citizen health insurance funds, parliamentarian, etc.). To foster a long-term vision on health financing, participants and experts recommended the development of a national charter on health financing. Box 5 presents the strategic recommendations for the governance function.

### The policy dialogue divergences

The debate in each workshop of the national conference and plenary sessions was organised through the following elements: presenting the point of view of experts (national and international), the moderator launches the general questions, and opens the debate. Moderators were given instructions to allocate more time for discussions and allow participants to express their views. However, the presence of health financing experts provided arbitration that reduced political divergence, especially when feasibility was analysed based on real international experiences. Despite that, disagreements concerning specific points (like the case of merging health insurance funds) were avoided by adjusting the recommendation through adopting a progressive merging in the long term rather than an immediate unification of funds. Another example is related to the fact that some actors suggested increasing reimbursement rates for health insurance schemes. The political sensitivity of this recommendation was mitigated by suggesting a revision of rates based on health technology assessment analysis so the increase could be objective. In this way, the purchasing system is strategic. The role of moderators was crucial to move from suggestions to adopted recommendations by confronting the expert point of view and invited resource persons from other countries. Facilitators were given the guidance to try to give the floor to all categories of participants and ensure the inclusion of all ideas.

### Workshops for drafting the strategy

To continue the coproduction process started before, within and after the national conference, the MoH, with the support of WHO, organised two workshops to identify concrete actions. The starting point was the conference recommendations. The participation of all technical departments in the coproduction process gave strength to the proposed measures. The objectives of the follow-up workshops were to sustain stakeholder involvement and increase the ownership of proposed actions, beyond the recommendations of the conference.

### The strategy draft

**Fig. 2:**
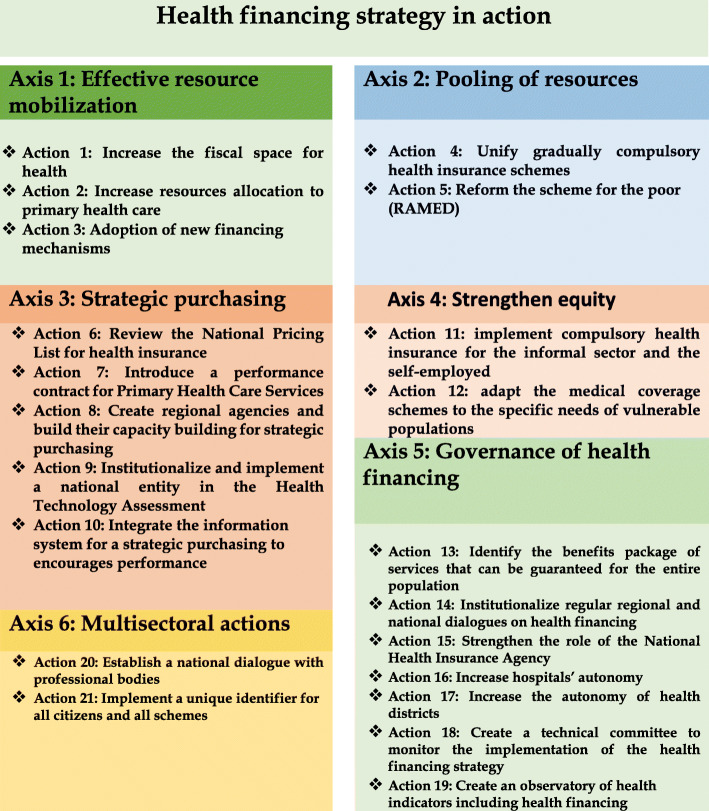
The main actions of the health financing strategy in Morocco

The draft strategy was developed along the following axes: (1) actions related to resources mobilisation, (2) the pooling of resources, (3) improving the purchasing function, (4) improving equity and ensuring the financial protection for vulnerable groups, (5) improving health financing governance, and (6) multisectoral actions related to health financing such as benefits package definition and health in all policies. Figure 2 presents the main axes and actions of the strategy draft.

Box 2 Main strategic recommendations for resources mobilisation in Morocco • Adopt and implement new financing mechanisms (e.g. sin taxes on products and goods that are harmful to health)

• Mobilise and prioritise resources for primary healthcare

• Accelerate the implementation of health insurance for self-employed workers with stable funding

• Increase fiscal space for health by strengthening tax revenue dedicated to health and strengthening the budget of the Ministry of Health

Box 3 Strategic recommendations for the Pooling function • Gradually work for the unification of compulsory health insurance schemes

• Ensure equity in funding by adopting the same contribution rate for all insureds and the same benefit package for a harmonisation of schemes and their convergence and fight against the fragmentation

• Encourage the pooling of risks, promote prepayment in order to reduce direct payments

• Establish a system of solidarity between schemes

Box 4 Strategic recommendations for the Purchasing function • Redefine a benefit package to be made available to the entire population, according to the needs of the population and evaluate feasibility, including funding, to deliver it to the whole population

• Define a body in charge of updating the benefits package and its mechanism based on priority-setting and health technology assessment tools

• Strengthen partnerships for processes of strategic purchasing

• Develop hospital autonomy by strengthening their management according to performance and self-financing

• Implement separation of financing and service provision functions

• Strengthen and set up an information system for monitoring the performance of healthcare structures (human resources component, funding and activity)

• Develop strategic purchasing methods based on performance contracting

• Orient the purchasing function to develop family medicine

• Develop the partnership to mobilise resources for health and encourage private investment in health

• Encourage the private sector to invest according to the need for health

• Strengthen the targeting system to allow financial protection of target and vulnerable groups

• Ensure that the service package and funding integrate the needs of vulnerable groups

Box 5 Strategic recommendations for the Governance function • Strengthen the governance missions of the National Agency of Health Insurance (*Agence Nationale de l’Assurance Maladie*) as the UHC regulatory authority

• Improve governance at regional level by setting up autonomous regional agencies and regional hospital groups

• Develop governance mechanisms based on information and intelligence (patient file, billing, etc.)

• Establish a national health charter

• Create governance bodies: inter-ministerial committee and regional health committees and institutional spaces for dialogue through the high Health Council

• Create regional and national dialogue forums

## Discussion

The objective of this article was to stress the central role of policy dialogue for developing a health financing strategy in Morocco and share this experience as a learning opportunity for other countries. In the past years, the development process of health strategies in Morocco adopted mostly a top-down approach with less involvement of all concerned actors. Former strategies on health financing were not implemented because of the lack of participation in their process and design and, thus, the lack of political legitimacy and technical ownership. In 2015, a strategy on health financing was developed by experts with suggestions of actions. This strategy could not find its way to becoming a formulated policy. The lack of involvement of parliamentarians, civil society, and the Ministry of Finance and the lack of high-level endorsement weakened the strategy adoption. Since 2011, the Arab spring social movement introduced a culture of participation as a way of legitimising policy decisions [36] but also empowered civil society and the population. The empowerment of policy actors was also seen in Tunisia with an interesting experience of policy dialogue for health system reform [37]. In Morocco, policy dialogue allowed a co-production approach to formulating the strategy and proved useful in the current experience of health financing. The steady mobilisation of policy-makers to support the debate increases the chances of successfully adopting and implementing strategic actions, thanks to alliance and consensus between decision-makers at the highest level. The national dialogue succeeded in mobilising all actors but, most importantly, kept their attention until the last day of debate through the momentum of follow-up workshops and co-writing the strategic actions. This dynamic demonstrates that national actors only need a space for discussion to contribute strategic ideas from their perspective. It is important to note that health financing in Morocco was designed since the beginning to give importance to the financial protection of citizens. Therefore, health services are free of charge in all public centres. Additionally, the scheme for the poor is the main component of the UHC model in Morocco, where the government is committed to providing health services for the poor. The sustainability of the financing, especially for the RAMED scheme, is one of the main challenges already known to have contributed to the success of this policy dialogue given the importance of health financing to achieve UHC.

The importance of dialogue in policy design appears to show its added value in other contexts, including LMICs. A case study from Liberia showed the role of policy dialogue to mobilise all stakeholders in supporting health policy design [23]. The authors also showed the importance of adequately conceptualising the dialogue to reach the desired objectives (choice of themes, facilitation methods, etc.). Finally, they concluded the importance of evidence to support the dialogue. Through our process, we also noticed the role of evidence to facilitate the policy dialogue along with a careful choice of facilitators and themes for discussions. The prominence of facilitators helped to avoid conflicts as a result of divergence in actors’ positions and opinions, especially when sensitive issues were raised. For credibility reasons, facilitators were selected from a range of known persons by their expertise and especially by their ability to achieve consensus (secretaries general, senior directors, parliamentarians, technical partners, senior experts). The fact that the policy debate on health financing did not confine itself to only a few financing elements was also a fundamental achievement. The role of the private sector was at the centre of the discussions either related to resource mobilisation or the purchasing function of health financing. It was mentioned that the preferred type of partnership is one that puts the population’s health at the centre beyond the sole aim of profit. The discussion during the conference explored specific issues related to the benefits package and suggested recommendations for the prioritisation and mechanisms to update its content. Other researchers discussed the benefits package and stressed the role of prioritisation and the agreement needed to set choices at that level [38, 39].

The literature on the benefits package definition shows the importance of involving stakeholders through debate and dialogue to set national priorities in terms of designing its content and the process to perform further adaptation [40]. Although the benefits package was a political and sensitive issue, participants did not shy away from discussing it and suggested that its current definition can no longer survive. With the current package, it is challenging to quantify and run studies to estimate the funding required to cover all the population. The national conference was an opportunity to simplify the health financing jargon and made it accessible to non-specialised actors. Different actors also had to learn from each other and share knowledge. This confirms previous assessments indicating that dialogue is a potential source of collective learning for organisations [41, 42]. Others stressed the place of strategic learning in the process of crafting strategies [43]. This paper presents a learning experience for other countries to improve the conceptualisation of policy dialogues not only in the health financing area but also for other health system aspects. The institutionalisation of the policy dialogue like the one on health financing came out of the discussions as an essential factor to strengthen policies in the future by ensuring extensive participation and mobilisation around national strategies.

The draft strategy proposed institutionalised dialogues at national but also at regional levels. For instance, existing examples of policy dialogue at the subnational level have proven effective in France (*conférences régionales de santé*) [44]. Another study conducted in Chad and Mali emphasised the need to institutionalise the policy dialogue to strengthen policies through the participation of all actors [21]. In the Moroccan setting, the institutionalisation of policy dialogues will create an accountability mechanism that will certainly enhance policy actions. The institutionalisation could be done through the organisation of periodic spaces for debate and creating a legitimised link between recommendations of dialogue and policy decisions.

This policy dialogue was the first experience in the area of health financing and is far from being perfect. It provides a first attempt to document this complex process and prepare the field for a more in-depth qualitative analysis of policy dialogues. The management of parallel sessions was a challenge because of the difficulty to simultaneously ensure freedom to participants to choose the sessions while keeping a representativity level in each session. Fortunately, the number of participants was more than expected (250 instead of 200), which allowed guaranteeing a good level of participation in each workshop. Maybe a prior pre-registration in workshops that respects an acceptable level of distribution and representativeness could solve the problem in the future.

The complexity of the process did not allow us to perform individual interviews to analyse the contextual determinants of the policy debate. It might have been interesting to explore the psychological influence of this experience on health actors and to what extent they are willing to support the implementation of the strategy in the long-term.

## Conclusion

With this article, we tried to provide a structured analysis of a fruitful policy dialogue on health financing. The paper presents an approach of crafting health strategies with more legitimacy through participation. Although implemented in other countries, this approach is fairly new in Morocco. We hope that further qualitative research will explore policy commitments for health financing and draw a picture of its determinants. The documentation of policy processes, especially those involving a participatory approach, will enhance the overall learning for UHC in Morocco and internationally.

## Data Availability

The documents related to the process of organising the policy dialogue are available under request to the first author.

## Abbreviations

LMICs: low- and middle-income countries
RAMED: *Régime d’Assistance Médicale*
UHC: Universal Health Coverage
